# C5b-9 Membrane Attack Complex Formation and Extracellular Vesicle Shedding in Barrett’s Esophagus and Esophageal Adenocarcinoma

**DOI:** 10.3389/fimmu.2022.842023

**Published:** 2022-03-08

**Authors:** Cathryn M. Kolka, Julie Webster, Ailin Lepletier, Clay Winterford, Ian Brown, Renee S. Richards, Wioleta M. Zelek, Yilang Cao, Ramlah Khamis, Karthik B. Shanmugasundaram, Alain Wuethrich, Matt Trau, Sandra Brosda, Andrew Barbour, Alok K. Shah, Guy D. Eslick, Nicholas J. Clemons, B. Paul Morgan, Michelle M. Hill

**Affiliations:** ^1^ QIMR Berghofer Medical Research Institute, Herston, QLD, Australia; ^2^ Envoi Pathology, Herston, QLD, Australia; ^3^ Division of Infection & Immunity, School of Medicine, Cardiff University, Cardiff, United Kingdom; ^4^ Centre for Personalised Nanomedicine, Australian Institute for Bioengineering and Nanotechnology (AIBN), The University of Queensland, St Lucia, QLD, Australia; ^5^ School of Chemistry and Molecular Biosciences, The University of Queensland, St Lucia, QLD, Australia; ^6^ University of Queensland Diamantina Institute, Faculty of Medicine, The University of Queensland, St Lucia, QLD, Australia; ^7^ National Health and Medical Research Council (NHMRC) Centre of Research Excellence in Digestive Health, Hunter Medical Research Institute, The University of Newcastle, Newcastle, NSW, Australia; ^8^ Cancer Research Division, Peter MaCallum Cancer Centre, Melbourne VIC, Australia; ^9^ Sir Peter MacCallum Department of Oncology, The University of Melbourne, Melbourne, VIC, Australia

**Keywords:** complement system activation, extracellular vesicle (EV), microvesicle, terminal complement component (SC5b-9), exosome

## Abstract

The early complement components have emerged as mediators of pro-oncogenic inflammation, classically inferred to cause terminal complement activation, but there are limited data on the activity of terminal complement in cancer. We previously reported elevated serum and tissue C9, the terminal complement component, in esophageal adenocarcinoma (EAC) compared to the precursor condition Barrett’s Esophagus (BE) and healthy controls. Here, we investigate the level and cellular fates of the terminal complement complex C5b-9, also known as the membrane attack complex. Punctate C5b-9 staining and diffuse C9 staining was detected in BE and EAC by multiplex immunohistofluorescence without corresponding increase of C9 mRNA transcript. Increased C9 and C5b-9 staining were observed in the sequence normal squamous epithelium, BE, low- and high-grade dysplasia, EAC. C5b-9 positive esophageal cells were morphologically intact, indicative of sublytic or complement-evasion mechanisms. To investigate this at a cellular level, we exposed non-dysplastic BE (BAR-T and CP-A), high-grade dysplastic BE (CP-B and CP-D) and EAC (FLO-1 and OE-33) cell lines to the same sublytic dose of immunopurified human C9 (3 µg/ml) in the presence of C9-depleted human serum. Cellular C5b-9 was visualized by immunofluorescence confocal microscopy. Shed C5b-9 in the form of extracellular vesicles (EV) was measured in collected conditioned medium using recently described microfluidic immunoassay with capture by a mixture of three tetraspanin antibodies (CD9/CD63/CD81) and detection by surface-enhanced Raman scattering (SERS) after EV labelling with C5b-9 or C9 antibody conjugated SERS nanotags. Following C9 exposure, all examined cell lines formed C5b-9, internalized C5b-9, and shed C5b-9^+^ and C9^+^ EVs, albeit at varying levels despite receiving the same C9 dose. In conclusion, these results confirm increased esophageal C5b-9 formation during EAC development and demonstrate capability and heterogeneity in C5b-9 formation and shedding in BE and EAC cell lines following sublytic C9 exposure. Future work may explore the molecular mechanisms and pathogenic implications of the shed C5b-9^+^ EV.

## Introduction

Recent studies have generated strong evidence of tumor-promoting actions of early complement components in the tumor microenviroment (TME) (1). The complement system is a protease cascade of the innate immune system of plasma proteins that triggers inflammation and helps immune cells to fight infections following activation by immune complexes, apoptotic cells (classical pathway) or foreign sugar motifs (lectin pathway). There is also a constitutive low sentinel activity *via* the alternative pathway. Each pathway leads to the cleavage of the central component C3 into bioactive fragments C3a and C3b, ultimately activating the terminal pathway *via* C5 cleavage into C5a and C5b (1). C3a and C5a are anaphylactic and chemotactic fragments involved in the recruitment of immune cells, while C3b and its degradation products are opsonins that facilitate phagocytosis. The C5b fragment binds with C6, C7, C8 and multiple units of C9 in the membrane, forming C5b-9, also known as the membrane attack complex (MAC), in the cell membranes of cells and microorganisms to cause cell lysis.

The early complement components C3 and C4, and the receptor C5aR have been reported as biomarkers for poor prognosis in gastric and colon cancers (2), with one mechanism being pro-tumor autocrine actions of C3a and C5a in the TME (3), including immune infiltration. In line with activation of the complement cascade, elevated plasma/serum C9 was reported for gastric (4) and colorectal cancer (5), although terminal complement activity and formation of C5b-9 in the cancer tissue was not examined.

Recently our group identified elevated C9 in the serum and tumors of patients with esophageal adenocarcinoma (EAC) (6, 7), a cancer of the gastro-esophageal junction associated with chronic gastro-esophageal reflux (GERD) (8–10). In agreement with our finding in patient cohorts, a rat model of gastroesophageal reflux showed differential expression of C9 mRNA during progression from the precursor condition Barrett’s esophagus (BE) to EAC (11). Furthermore, complement components C3 and C1r were also elevated in serum collected from high grade dysplastic BE and EAC patients (12), and C1q mRNA (13) and protein has been detected in BE and in tumors of EAC, likely in macrophages and dendritic cells (14). As non-classical complement-associated role of C1q has been suggested in other cancers (15), it is important to establish whether the observed elevated complement component proteins in EAC leads to terminal complement activity (C5b-9/MAC formation). Furthermore, while we observed strong or moderate C9 staining in dysplastic BE and moderate C9 staining in EAC tissues, respectively (6, 7), the C9-positive BE/EAC cells appeared to be morphologically healthy and not subjected to complement-mediated cell lysis. Cancer cells are known to resist complement-induced cell lysis by clearing C5b-9 from the cell surface *via* endocytosis or exocytosis (16). In this study, we examined the formation and fates of C5b-9 across the stages of EAC development using tissues and cell lines, and also investigated the source of the elevated C9 in the tissue.

## Methods

### Patient Cohorts for Immunohistochemistry

Two tissue microarrays were analyzed in this study. Information on the EAC tissue microarray was previous published (17, 18). A BE tissue microarray was generated from formalin-fixed, paraffin-embedded archival esophageal tissue samples retrieved from 89 patients at Envoi Pathology, Brisbane, Australia. This project was approved by the QIMR Berghofer Medical Research Institute Human Research Ethics Committee (P2352). No patients received pre-operative chemotherapy or radiotherapy.

### RNA Sequencing

RNA sequencing was performed on 9 BE, 8 EAC and 7 matched healthy esophageal (NE) tissue samples collected from participants at the Princess Alexandra Hospital, Brisbane, Australia, with written informed consent. Ethics approval was granted by the Princess Alexandra Hospital and QIMR Berghofer Medical Research Institute research ethics committees (PAH-HREC-2007/068, HREC/10/QPAH/152, HREC/11/QPAH/529 and QIMRP514). The samples were collected and stored in RNAlater prior to RNA extraction and sequencing. RNA samples were extracted using previously published methods (19) and were analyzed using the Illumina HiSeq 2500 high-throughput sequencing system (Illumina, Inc., San Diego, USA). STAR aligner (version 2.5.2a) was used to align the paired-end reads to the human reference genome cersion GRCh37. Cutadapt (version 1.9) was used to trim the sequence adaptors and gene expression was estimated using RSEM (version 1.2.30). Trimmed mean of M values (TMM) normalization was applied to the expression data using the R package edgeR.

### Gene Expression Datamining

Gene expression array data was obtained from Gene Expression Omnibus accession number GSE72874 (20). Publicly available data of 78 EAC and 9 NE were obtained from the TCGA Data Portal (https://portal.gdc.cancer.gov/), downloaded as fully processed level 3 data. TMM normalization was applied to the expression data using the R package edgeR.

### Immunohistochemistry (IHC)

Archival formalin fixed paraffin embedded (FFPE) tissue microarrays were sectioned at 3µm on superfrost+ slides. Sections were dewaxed in xylene, rehydrated in descending concentrations of ethanol to water and endogenous peroxidase quenched with 2% H_2_O_2_ in pH 7.5 Tris-buffer saline for 10 min. Antigen retrieval was carried out in a Biocare Medical Decloaking Chamber using Dako Target Retrieval Solution pH 9 for 15 min at 105°C. To eliminate nonspecific background staining, sections were blocked with 3% hydrogen peroxide in tris-buffered saline with 0.01% Tween-20 (TBS-T) for 5 minutes and Biocare Medical Background Sniper in 2% BSA for 15 min.

For chromogenic staining, Sigma Rabbit anti-human C9 primary antibody (Sigma Aldrich #SAB4503059) was diluted 1:300 in Background Sniper + 2% BSA for 60 minutes at room temperature. The specificity of this C9 antibody in IHC was previously assessed (7) by incubation of the antibody with recombinant purified C9 prior to using the antibody for IHC. Staining was visualized using Biocare Medical MACH2 Rabbit Polymer HRP detection system applied for 30 minutes, and Betazoid DAB for 5 minutes. Sections were washed, and counterstained in Mayers’ haemotoxylin, dehydrated through ascending graded alcohols, cleared in xylene, and mounted using DePeX. IHC images were obtained in Aperio AT turbo digital slide scanner from Leica Biosystems.

### Multiplex Immunohistofluorescence (mIHF)

Chromogenic images are typically used for pathology scoring; however, they are unsuitable for colocalization of overlapping markers, therefore we developed a simultaneous mIHF protocol for C9 and C5b-9 staining in archival-FFPE tissue specimens. 3µm slides were deparaffinized, rehydrated, and washed in TBS-T. Antigen retrieval was performed in Dako Target Retrival Solution pH 9 for 15 min at 105°C. All multiplex steps were with two TBS-T washes between each step. Prior to staining, tissue sections were blocked with 3% hydrogen peroxide in TBS-T for 5 minutes and background sniper for 10 minutes (Biocare Medical). Sequentially staining for C9 and C5b-9 negatively impacted on the staining of the second stain. By applying antibodies simultaneously and labelling them sequentially, both signals were maintained when compared to the single stains, validated on human esophageal carcinoma sections. For simultaneous staining, primary antibody Sigma C9 rabbit polyclonal (Sigma #4503059) diluted 1:1000 and C5b-9 mouse monoclonal clone aE11 (Abcam #ab66768), targeted against a C9 neoepitope, diluted 1:2000 was applied for 30min at room temperature. For visualization of C9 staining, after washing, Biocare Medical MACH2 Rabbit Polymer HRP was applied for 15minutes following 10 minutes incubation with tyramide signal amplification (TSA)-based fluorophores AKOYA Opal 520 1:100 in Opal 1xPlus Amplification Diluent for 10 minutes. For visualization of C5b-9 staining, sections were washed, MACH2 mouse polymer HRP applied for 5 minutes, then incubated with TSA-based fluorphore AKOYA Opal 620 1:100 in Opal diluent for 10 minutes. Sections were counterstained with DAPI (5mg/ml 1:35000 in TBS) for 5 minutes, washed, and coverslipped using Dako Fluorescence Mounting Media.

mIHF images were obtained using Vectra 3.0 automated quantitative pathology system (Perkin Elmer). Fluorescent whole slide scan at 4x magnification was produced and visualized in Phenochart (v1.04), followed by multispectral image acquisition of each tissue microarray (TMA) core or selected high power regions at 20x magnification. Multispectral images were spectrally unmixed using InForm analysis software (v2.2.1) from Perkin Elmer.

### Scoring of TMA

Biopsies were classified as normal squamous epithelium (NSE), Barrett’s esophageal mucosa (BE), dysplasia (low and high grade) (LGD and HGD), intraepithelial carcinoma (IEC) and EAC by a specialist gastrointestinal pathologist, and staining intensities for C9 and C5b-9 were scored. When the staining was non-uniform, the maximum intensity of staining in the epithelium was used for the score, providing at least 10% of the cells of that component stained to this intensity. Each component, if present in the tissue, was scored separately using a 4-grade assessment of intensity (0 no staining, 1+ weak staining, 2+ moderate staining, 3+ strong staining). Each cohort used liver as a positive C9 control for normalization between cohorts/slides.

Colocalization between C9 and C5b-9 was determined using InForm Advanced Image Analysis software (Perkin Elmer), which provided a percentage of the C9 positive area that was also positive for C5b-9, and the C5b-9 positive area that was also positive for C9. Nonspecific autofluorescence signal from red blood cells was removed using Inform software, and threshold of detection was set visually for the whole TMA.

### Cell Culture

Non-dysplastic BE and dysplastic esophageal cell lines CP-A, CP-B and CP-D were provided by Dr P. Rabinovitch (University of Washington, USA); BART-T cells were provided by Dr Rhonda Souza (Baylor University Medical Center, TX). All non-dysplastic BE and dysplastic esophageal cells were cultured in Keratinocyte Serum Free Medium (KSFM) in 2.5% heat inactivated fetal bovine serum (HI FBS) (Gibco), supplemented with 0.1 μg/mL Epidermal Growth Factor (PeproTech) and 0.7 μg/mL of Bovine Pituitary Extract (Sigma). OE33 and FLO-1 were purchased from the European Collection of Authenticated Cell Cultures (Sigma). OE33 were cultured in Roswell Park Memorial Institute 1640 medium (RPMI) in 10% HI FBS, while FLO-1 were cultured in Dulbecco’s modified Eagle’s medium (DMEM) in 2.5% HI FBS. All cell lines were cultured in a 5% CO2 incubator set to 37°C. FBS used throughout this study was heated to inactivate complement components.

### C9 Treatment of Cultured Cells

Immunopurification of C9 from human serum, generation of C9-depleted human serum and their detailed characterization were previously reported (21). Cells were seeded on to coverslips. After approximately 2 days growth to ~60% confluence, medium was removed and cells incubated in serum-free medium (KSFM, DMEM or RPMI, according to cell requirements) containing 0.1% BSA for 4 hours to remove any complement components remaining after heat inactivation. C9 treatment was conducted at 37°C, 5% CO_2_ in medium containing 10% C9-depleted human serum, with/without addition of 3µg/ml immunopurified C9. This dose was chosen to be lower than a previously published lytic dose of 20µg/ml in K562 cells (16), as well as the reported serum concentration of C9 in BE and EAC patients (10-15 µg/ml) (21). 3µg/ml of immunopurified C9 did not cause substantial cell lysis in our experimental set up, determined by lactate dehydrogenase assay in 3 cell types.

After selected exposure times up to 120 minutes, medium was harvested to tubes on ice, then centrifuged at 1500 g 4°C for 15 minutes. The supernatants were collected and stored at -80°C for extracellular vesicle analysis. Coverslips with cells were immediately washed with ice cold PBS, then fixed with 4% paraformaldehyde at room temperature for 20 minutes. For immunofluorescence, coverslips were blocked and permeabilized with 0.3M glycine and 0.1% Triton X-100 at room temperature for 20 minutes then labelled with the indicated primary antibodies: C5b-9 (abcam ae11, 1/500), LAMP-1, (rabbit: LAMP-1: Abcam ab24170), then washed, labeled with secondary antibodies rab-647 and m-488 (1/300), then washed and stained with DAPI (1/1000). Labelled cells were analyzed under a Zeiss 780 NLO Microscope (Oberkochen, Germany), with Z-stack analysis with each slice at approximately 0.38µm on 63x or 100x objective, image size of 1024x1024. Images and merged images were obtained with Zen software (Carl Zeiss, GmbH, Germany). Images were processed further for display by using ImageJ (National Institutes of Health). Maximum intensity projection images were thresholded consistently for all images, C5b-9 puncta with sizes over 10 pixel^2^ were included for particle analysis, and total area calculated per image: these values were divided by the number of cells in the image to provide puncta/cell and positive C5b-9 area/cell.

### Incucyte

Cells were plated in 96 well tissue culture plate in appropriate media with depleted serum, with or without 3µg/ml C9. Propidium Iodide (15nM final concentration) was included to assess dead cell number. Plates were assessed in the Incucyte (Incucyte Zoom, Sartorius) for up to 80 hours, 5% CO_2_ and 37°C, with images taken every 4 hours, and images processed using Incucyte software to detect confluence and red object count as an indication of the number of dead cells.

### Extracellular Vesicles (EV) Analysis

C9+ and C5b-9+ EV analysis was performed using an immunoassay integrated on a microfluidic device with asymmetric gold electrodes and surface-enhanced Raman scattering (SERS) read-out, as previously reported (21). The electrodes of this SERS assay were functionalized with anti-CD9/CD63/CD81 antibodies (CD9: clone 5G6; CD63: clone H5C6; CD8: clone 1D6; Novus Biologicals) to capture EVs in the sample. Subsequently, the captured EVs were labelled with SERS nanotags that targeted C9 (C9 rabbit polyclonal Sigma #4503059) or C5b-9 (abcam ae11) expression. The SERS nanotags were spherical gold nanoparticle that were functionalized with antibodies against C9 or C5b-9 and Raman reporters (5,5′-dithiobis 2-nitrobenzoic acid (DTNB) for C9 and 2,3,5,6-tetrafluoro-4-mercaptobenzoic acid (TFMBA) for C5b-9). The average Raman shifts of the DTNB peak at 1335 cm^-1^and TFMBA peak at 1335 cm^-1^ were used for quantification of C9 and C5b-9, respectively.

50 µL of cell culture supernatant sample and 20 µL SERS nanotags were sequentially added to each well of the microfluidic device. Following previously optimized conditions (22), alternating current was applied for sample incubation (i.e., frequency = 500 Hz, amplitude = 800 mV, incubation time = 45 min) and for labelling with SERS nanotags (500 Hz, 800 mV, 20min), respectively. The alternating current induced a nanoscopic fluid flow on the surface of the antibody-functionalized asymmetric gold electrodes to stimulate EVs capture and labelling. After incubation, the asymmetric gold electrodes were washed with 1% BSA in PBS three times. The microfluidic device was stored at 4°C prior to SERS mapping. SERS mapping was performed using a WITec Alpha 300 R confocal Raman microscope (Ulm, Germany). A 20× microscopic objective and HeNe laser (32 mW, 633 nm) were utilized. The parameters used for SERS mapping were 0.25 s integration time, 1µm step size, and 60 μm × 60 μm (60 pixel × 60 pixel) mapping area. Initially, the Raman microscope was calibrated by measuring the silicon substrate signal intensity at 520 cm^-1^. The samples were run in triplicate. The raw Raman spectral data contained fluorescence and background noise that was removed using a fifth-order polynomial fitting method previously described [23].

## Results

### Tissue C9 and C5b-9 Staining Increase Through the Stages of EAC Development

To evaluate esophageal tissue C9 and C5b-9 levels during the sequence normal epithelium (NE)-BE-EAC, we generated and stained a tissue microarray (TMA) from archival esophageal tissue samples from patients undergoing investigation for BE or suspected EAC, and also stained a previously established TMA of EAC cases (17, 18). A multiplex immunohistofluorescence (mIHF) method was developed to enable co-staining and co-localization of C9 and C5b9, as detailed in Methods. For the purpose of cross-validating our previous data, the chromogenic C9 staining protocol used in our previous study (6, 7) was performed on a consecutive TMA section and showed the same trend of increasing score with disease stage as previously reported (**Figure S1**). Furthermore, direct comparison of chromogenic and immunofluorescent images on slices from the same biopsy demonstrates the similar pattern of staining (**Figures S1A**, **1**) and the trend in scores (**Figures S1B**, **1G**). Therefore, we were satisfied that the mIHF co-staining accurately reflects C9 levels.

**Figure 1:**
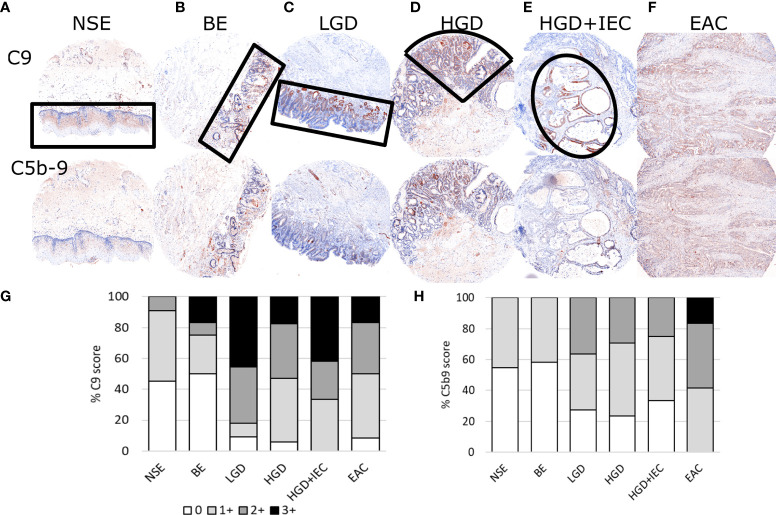
Representative immunohistofluorescence for C9 and C5b-9 in esophageal tissues during stages of esophageal adenocarcinoma development. Tissue microarrays of esophageal biopsies and esophageal adenocarcinoma (EAC) specimens were stained for C9 and C5b-9 using multiplex immunohistofluorescence, and then exported as chromogenic images for visualizations and scoring. Representatives images for the stages of EAC development are shown in panels **(A–F)**, with the areas marked by black lined shapes: normal squamous epithelium (NSE), non-dysplastic Barrett’s esophagus (BE), low-grade dysplasia (LGD), high-grade dysplasia (HGD), HGD with intraepithelial carcinoma (HGD+IEC) and EAC. Cores are 1.5mm. The whole EAC section is cancerous. Staining intensity was scored for C9 **(G)** and C5b-9 **(H)** for each tissue phenotype on a 0-3 scale by a specialist pathologist.

TMA sections co-stained for C9 and C5b-9 were evaluated for staining intensity in regions of specific tissue phenotypes (NSE: normal squamous epithelium, BE: Barrett’s Esophagus, LGD: Low grade dysplasia, HGD: high grade dysplasia, HGD+IEC: HGD with intraepithelial carcinoma, EAC) using a semi-quantitative scoring system from 0 to 3 by a specialist gastrointestinal pathologist (**Figure 1**). For the purpose of visualization, the fluorescent images were converted to the traditional chromogenic colors using Inform software. The normal squamous epithelium (**Figure 1A**) shows some staining for C9 but very little C5b-9. The glandular epithelium in BE, LGD, HGD and HGD+IEC shows high staining for C9, which was often associated with similar C5b-9 staining (**Figures 1B–E**). Finally, diffuse C9 and C5b-9 staining was observed in EAC samples (**Figure 1F**).

The quantitative result shows increased intensity and proportion of both C9 and C5b-9 staining with disease stage (**Figure 1G**), with effect likelihood tests of 0.0002 for C9, and 0.0004 for C5b-9. Furthermore, ordinal logistic regression for consecutive classifications showed a significant change from BE to LGD in both C9 (p=0.0008) and C5b9 (p=0.0306). The overall statistical analysis indicated a correlation between high C9 deposition and C5b-9 formation with disease stage.

**Figure 2:**
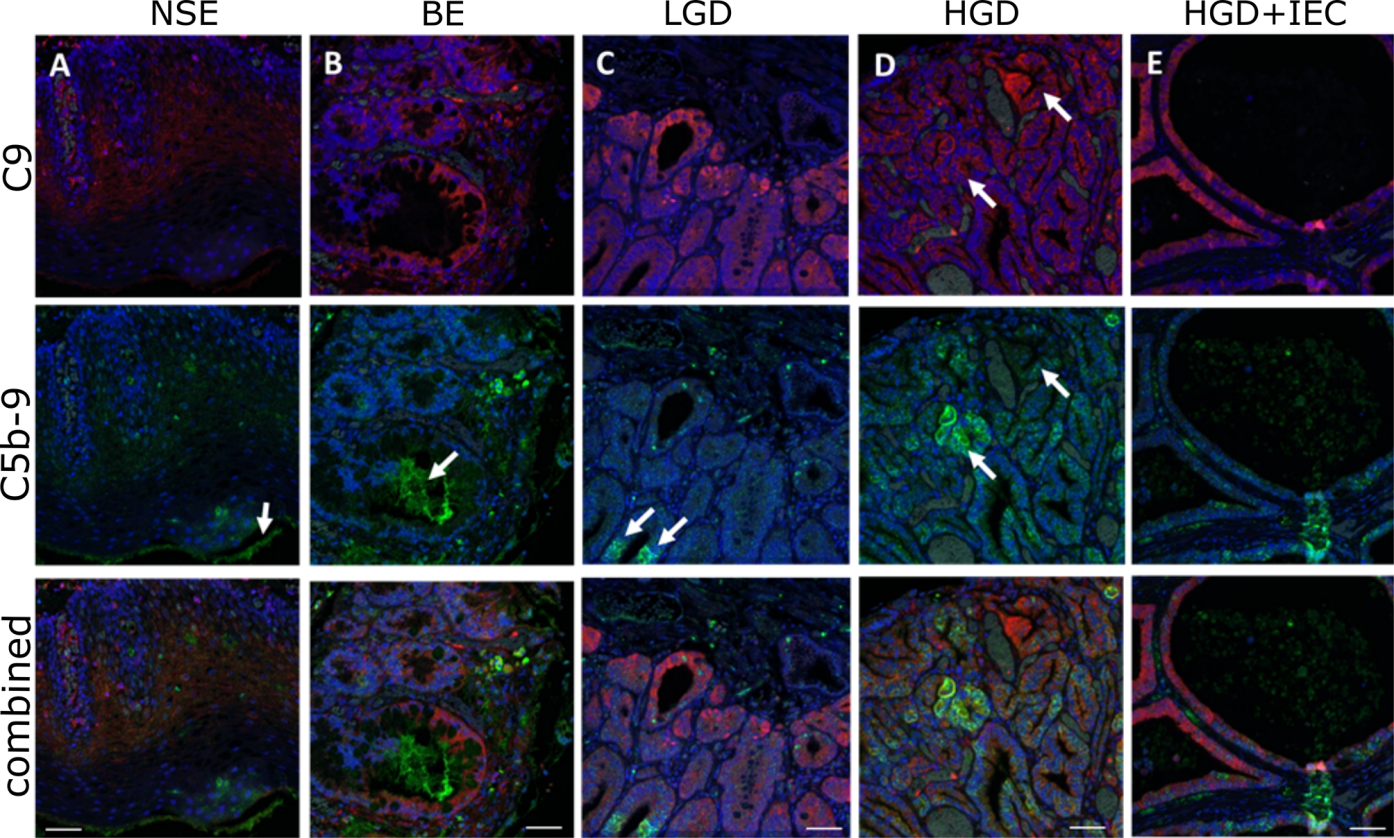
Localization of C9 and C5b-9 in esophageal tissues during stages of esophageal adenocarcinoma development. High magnification images of the multiplex immunohistofluorescence staining for C9 (red) and C5b-9 (green), with the blue DAPI stain for nucleus. **(A)** NSE, **(B)** BE, **(C)** LGD, **(D)** HGD, **(E)** HGD+IEC. Arrows in **(A, B)** highlight areas of C5b-9 staining that are not associated with nuclei. Arrows in **(C)** show areas positive for C5b-9 that are associated with nuclei and are likely cellular: these cells are weakly positive for C9. Arrows in **(D)** highlight two areas with similar C9 deposition, but different C5b-9 formation: C9 positive cells are not always strongly positive for C5b-9. E demonstrates the strong epithelial C9 staining in cystically dilated glands. Scale bar is 50µm.

Next, we used the high-resolution mIHF images to examine whether the C9 and C5b9 staining co-localized in the same tissue component and cell. C9 is typically strongly stained in the epithelium. C5b-9 is also located in the epithelium, however areas positive for C9 are not always positive for C5b-9 (**Figure 2**). Staining for C5b-9 can be punctate, particularly in cellular areas, likely reflecting clusters of MAC pores (**Figure 2**). Quantification of the colocalization of C9 and C5b-9 for each tissue phenotype revealed ~20% of C5b-9 co-stained for C9, and ~10% of C9 co-stained for C5b-9, with no differences observed between disease stages (**Table S1**).

Based on these findings, we postulated that the differential localization of C9 and C5b-9 could be explained if C9 is being expressed in the epithelial/cancer cells, while the C5b-9 is being deposited as MAC. To investigate C9 transcript expression, we made use of the transcriptome data in The Cancer Genome Atlas (EAC *vs* normal), as well as our own RNAseq dataset of normal esophageal epithelium, BE and EAC tissues (**Figure S2**). No C9 transcript was detected in normal esophageal tissue, and no significant change was detected in either dataset although C9 transcript was detected in some BE and EAC tissues at low levels and with a broad spread (**Figure S2**).

### C9 Exposure Induces Variable C5b-9 Formation and Internalization in BE and EAC Cells

The tissue immunohistochemistry data confirm the formation of C5b-9 on BE and EAC tissues. As the tissue morphologies are grossly intact, we hypothesize that BE and EAC cells resist complement-mediated cytolysis. MAC can be cleared from cells through endocytosis or shedding (16). To investigate C5b-9 clearance mechanism in BE and EAC, we used representative cell lines in an *in vitro* MAC formation experimental setting where a sublytic dose of immunopurified human C9 was added to culture medium containing C9-depleted human serum followed by C5b-9 detection by immunofluorescence. Two cell lines each were used for the disease stages of non-dysplastic BE (Bar-T and CP-A), HGD (CP-B and CP-D), and EAC (FLO-1 and OE-33). With the commercially available HET-1A cell line reportedly lacking characteristics of normal esophageal squamous epithelial cells (24), limited access to primary esophageal squamous cells of sufficient quantity, and the observed similar C5b9 staining of NSE and non-dysplastic BE (**Figure 1G**), we used the 2 available non-dysplastic BE cell lines as control.

**Figure 3:**
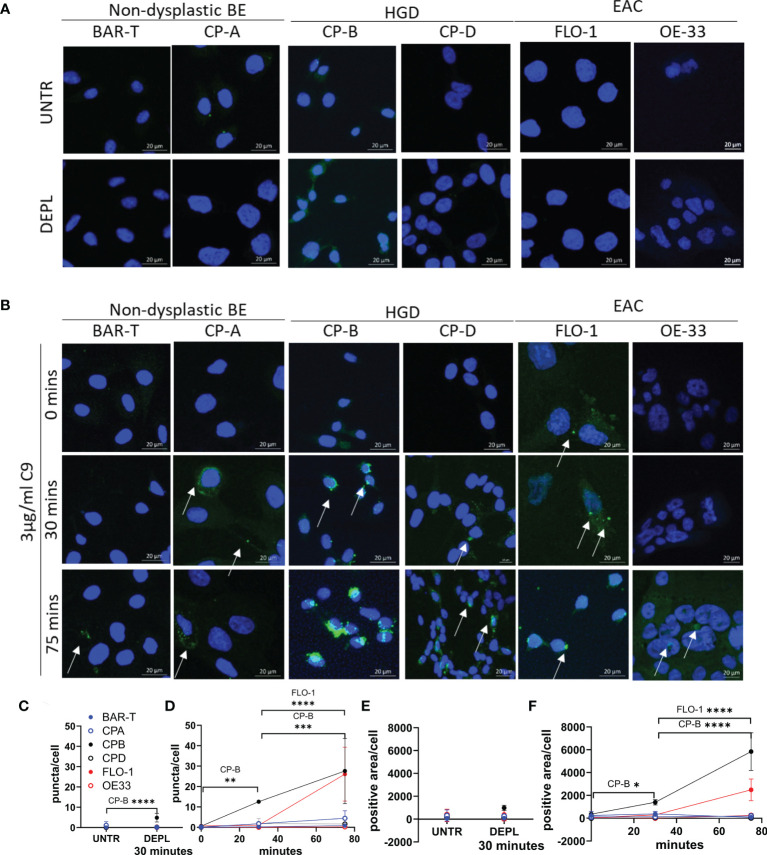
Time course of C5b-9 puncta formation in Barrett’s esophagus and esophageal adenocarcinoma cell lines after exposure to immunopurified C9. Cell lines representing non-dysplastic BE (BAR-T, CP-A), high-grade dysplasia (CP-B, CP-D) and EAC (FLO-1 and OE-33) were incubated in serum-free medium for 4 hours before being incubated in medium containing C9-depleted human serum for 30 minutes **(A)**, or medium containing C9-depleted human serum plus 3 µg/ml purified human C9 for 0, 30 or 75 minutes **(B)**. Coverslips were removed and stained with C5b-9 antibody and imaged by confocal microscopy. Maximum intensity projection of C5b-9 signal at 0, 30 and 75 min in all cell lines, cell nuclei are represented by blue (DAPI) and C5b-9 by green. Images are all at the same magnification. All images (**Figure S4**) were used to quantify the average number of puncta per cell **(C, D)** and the area positive for C5b-9 per cell **(E, F)** in untreated or cells incubated for 30 minutes in C9 depleted human serum **(C, E)**, or with C9 for 0, 30 or 75 minutes **(D, F)**. *P<.05. **P<0.01. ***P<0.001. ****P<0.0001.

Prior to the experiment, cells were washed and incubated in serum-free medium for 4 hours in order to remove bovine C9 (from serum) from the cells. At various time points following immunopurified human C9 treatment in the presence of C9-depleted human serum, cells on coverslips were fixed and then stained for C5b-9. Control conditions include untreated cells and cells exposed to C9-depleted human serum for 30 minutes (**Figure 3A**) showed no or low level C5b-9 puncta. Following treatment with immunopurified human C9, all 6 cell lines exhibited formation of MAC as C5b-9 puncta, but at substantially different levels despite the same dose of C9 exposure (**Figures 3**, **S3**). While both non-dysplastic BE cell lines showed low C5b-9 puncta, HGD and EAC cell lines exhibited variable formation and accumulation. The number of C5b-9 puncta per cell was quantified across all 6 cell lines and time points, confirming CP-B cell line has the highest C5b-9 at 30 minutes, with both CP-B and FLO-1 cells showing apparent high intracellular accumulation at 75 minutes (**Figures 3C, D** and **Table S2**). Quantification of C5b-9 puncta includes all puncta over 10pixel^2^, which likely underestimates the number of puncta in CP-B, where overlapping or adjoining puncta are read as a single spot, highlighting the striking amount of C5b-9 in CP-B. An alternative method of quantitation using the area positive for C5b-9 in each image divided by the number of cells highlights that both CP-B and FLO-1 demonstrate a substantial increase in C5b-9 (**Figure 3E, F**): in this case CP-B has a higher C5b-9 positivity than FLO-1, with significantly higher results at both 30 (P=0.0452) and 75 minutes (P<0.0001).

**Figure 4:**
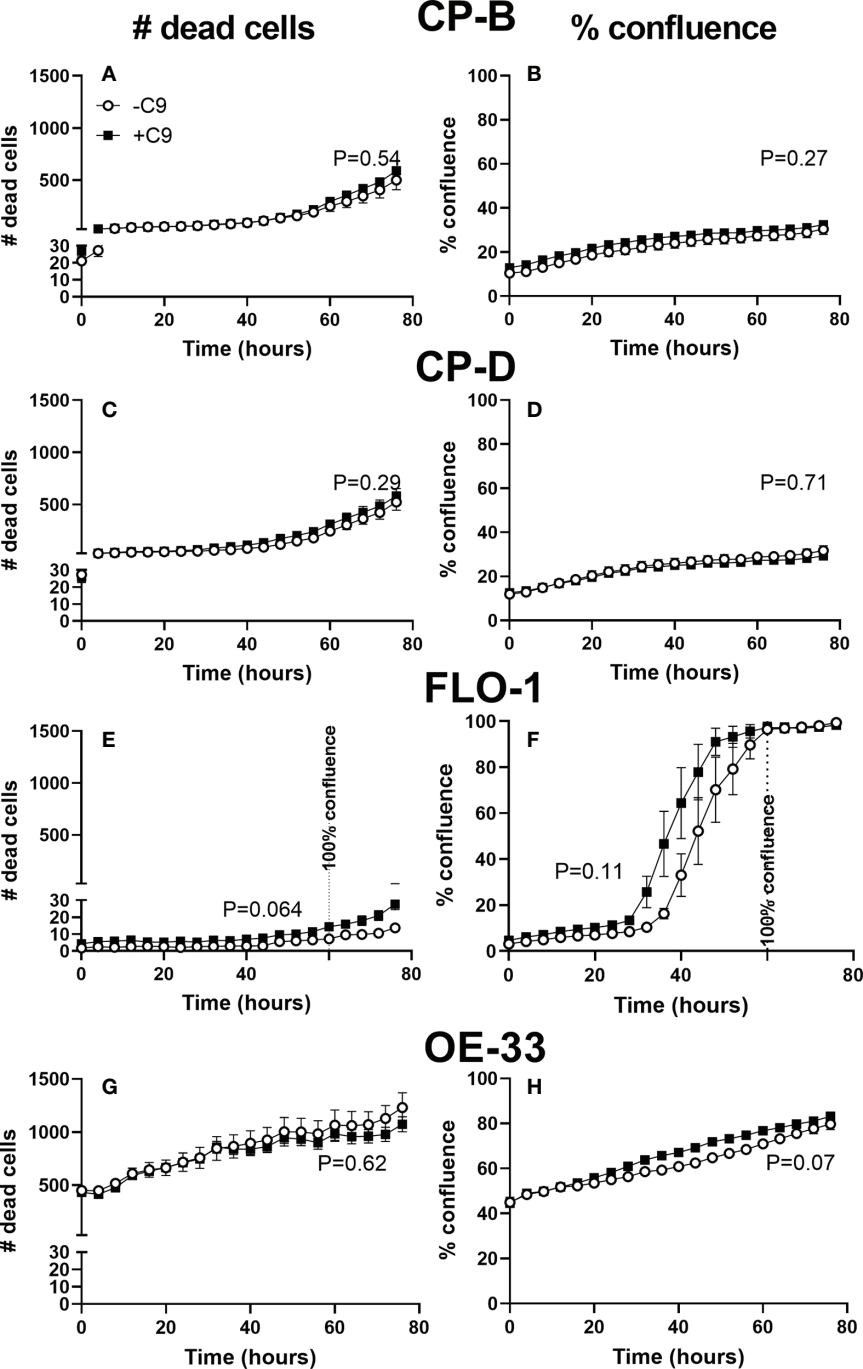
Cell proliferation and death of esophagus and esophageal adenocarcinoma cell lines with and without C9 exposure. Cultured CP-B **(A, B)**, CP-D **(C, D)**, FLO-1 **(E, F)** and OE33 **(G, H)** cells were seeded into 96 well plates with and without C9 present (3µg/ml). Incucyte time-lapse videos were used to capture cell proliferation **(B, D, F, H)** and death **(A, C, E, G)** (by propidium iodide staining). Two way ANOVA with Sidak’s multiple comparison test was performed, and showed no significant effect of C9 on either proliferation or cell death (p values for C9 effect shown in figure). FLO-1 cells reach 100% confluence by 60hr, indicated by vertical line. No other cell lines reached confluence during the experiment.

In spite of the MAC formation, C9 exposure did not lead to significant cell death up to 4 days in CP-B, CP-D, FLO-1 or OE33 cells (**Figure 4**). FLO-1 cells rapidly reached confluence at approximately 60 hours, and the trend toward increased cell death (P=0.064) appears to occur only after confluence is reached. In addition, the total amount of cell death in FLO-1 is low compared to all other cell lines. No other cell line approaches confluence, and the presence of C9 had no significant effect on the number of dead cells (CP-B, P=0.54. CP-D, P=0.29. FLO-1, P=0.064. OE33 P=0.62), or cell growth (CP-B, P=0.27. CP-D, P=0.71. FLO-1, P=0.11. OE33 P=0.07) in any cell line (two-way RM ANOVA with Sidak’s multiple comparison test).

### C5b-9 Release in Extracellular Vesicles from BE and EAC Cells

**Figure 5:**
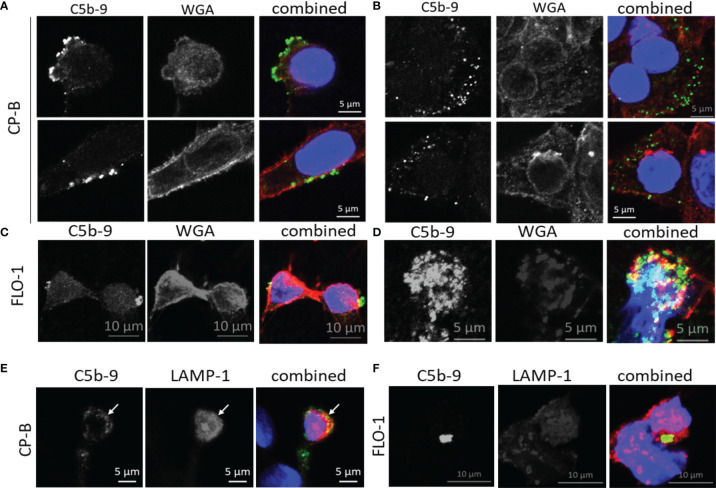
Subcellular localization of C5b-9 in Barrett’s esophagus and esophageal adenocarcinoma cell lines after exposure to purified C9 in culture. After 75 minutes of C9 exposure of CP-B **(A, B)** or FLO-1 **(C, D)** cells as described for **Figure 3**, co-staining of C5b-9 (green) was conducted with wheat germ agglutinin (WGA, red) to visualize the plasma membrane. The nucleus was stained with DAPI (blue). C5b-9 was observed at the cell surface **(A, C)**, as well as intracellularly **(B, D)**. These images represent one slice of Z-stack images acquired by confocal microscopy, adjoining Z-stack images are shown in **Figure S4**. A separate experiment with 120 minutes of C9 exposure was used for co-staining with the lysosome marker LAMP-1 (red) in CP-B **(E)** and FLO-1 **(F)**. Yellow color in the combined images indicates colocalization between the red and green signals.

In other cell types, assembled MAC can be cleared from the plasma membrane *via* endocytosis for lysosomal degradation (25), and shedding *via* EV (16), however, no studies have been performed on BE or EAC cells. As a first step, we conducted co-localization studies using 2 cell lines with the highest C5b-9 puncta from the time-course study, CP-B and FLO-1 (**Figure 5**). Using wheat germ agglutinin to label the cell surface, C5b-9 puncta could be observed at membrane blebs on the cell surface (**Figure 5A**) and intracellularly (**Figure 5B**, consecutive Z-stack images are shown in **Figure S4**). Some co-localization with LAMP-1 (lysosome marker, **Figures 5C** **, D**) could be observed, in agreement with previous data for other cells. We were intrigued that shedding of MAC-decorated EVs from BE and EAC cells could be a potential mechanism of our initial observation of increased serum C9 (7, 21). Therefore, we next aimed to quantify MAC-decorated EVs from cell culture supernatant.

**Figure 6:**
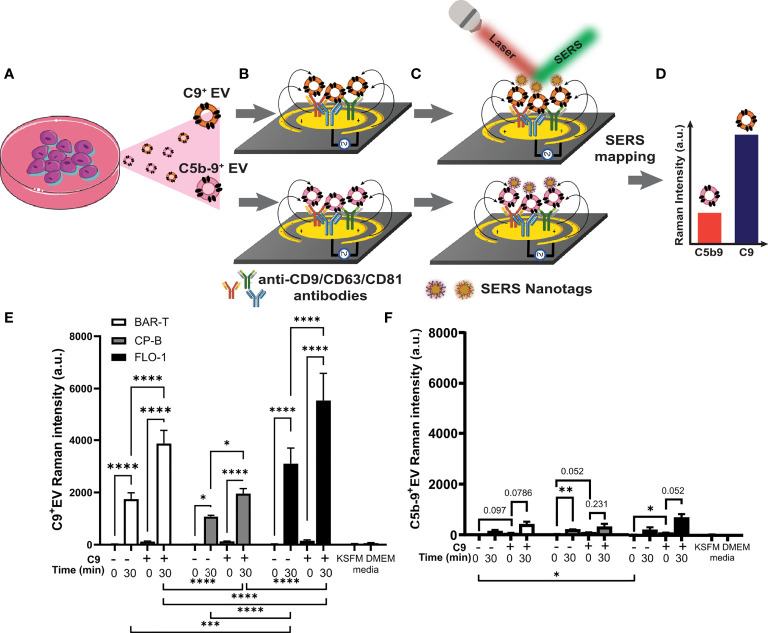
Barrett’s esophagus and esophageal adenocarcinoma cell lines release extracellular vesicles containing C9 and C5b-9 after exposure to C9. BAR-T, CP-B and FLO-1 cells were exposed to C9-depleted serum plus 3 μg/ml C9 for 0 or 30 min as described in **Figure 3**. The medium were collected and ab EV-capture SERS immunoassay performed to analyze C9 and C5b-9 on EV. **(A–D)** Schematic of EV-SERS assay: **(A)** Conditioned cell culture medium is added to **(B)** anti-CD9/CD63/CD81 antibody functionalized microelectrodes to capture C9^+^ and C5b-9^+^ EVs. **(C)** Captured EVs are detected with SERS nanotag-labelled anti-C9 or anti-C5b-9 antibody. In **(B, C)**, the application of an alternating current electric field on the microelectrodes induces a nanoscopic fluid flow (black arrow) that stimulates collisions of EVs with antibody-functionalized microelectrode surface. **(D)** SERS mapping reveals the expression levels of C9 and C5b-9 on EVs. **(E, F)** SERS assay measured extracellular vesicles that are positive for C9 **(E)** or C5b9 **(F)**, n=3. Controls include conditioned medium from cells exposed to C9 depleted serum (DEPL) for 30 minutes without addition of C9, as well as unconditioned medium (KSFM and DMEM) with depleted serum measured by 2 way ANOVA and Tukey’s multiple comparison test (*P < 0.05, **P < 0.01, ***P < 0.001, ****P < 0.0001).

We previously reported the capture of intact EVs from conditioned medium using microfluidic immunoassay using surface enhanced Raman spectroscopy (SERS) read-out of EV surface markers (26). Here, we adapted this sensitive technique to measure EVs with C5b-9 or C9 on the EV surface, using 3 tetraspanin antibodies (i.e., CD9, CD63, CD81) to capture EVs from conditioned media (**Figures 6A** **, B**) and SERS nanotag conjugated anti-C5b-9 (or anti-C9) antibodies to measure C5b-9^+^ (or C9^+^) EVs (**Figure 6C**), detected by SERS mapping (**Figure 6D**). To accommodate the limited reagents, one cell line was chosen for each pathological state: non-dysplastic BE (BAR-T), HGD (CP-B) and EAC (FLO-1) in this proof-of-concept study. Western blotting was performed to ensure that there was no detectable C9 in cell conditioned medium without treatment (**Figure S5**).

Each cell line was treated with C9-depleted human serum alone or with addition of immunopurified human C9. The treatment medium was immediately collected as control (labeled 0 min in **Figure 6E** **, F**), or the cell with treatment incubated for 30 minutes at 37°C. In addition, untreated medium without serum (KSFM, DMEM) served as negative control to assess the assay signal noise (**Figure 6E** **, F**). While the data for C9^+^ EV (**Figure 6E**) and C5b-9^+^ EV (**Figure 6F**) show similar overall patterns for the 4 experimental conditions, much stronger Raman signals were observed for C9^+^ EV over C5b-9^+^ EV. As shed MAC should be detected by both C9 and C5b-9 antibodies, we assumed the difference in intensity is due to different antibody efficacy. Other potential interpretations are explored in the Discussion section.

For all 3 cell lines, no C9^+^ EV was detected in C9-depleted serum alone at 0 min (**Figure 6E**), confirming that lack of C9^+^ EV within the depleted serum. Incubation with C9-depleted serum alone for 30 minutes led to significant shedding of C9^+^ EV (**Figure 6E**), presumably due to the trace residual C9 in the C9-depleted human serum (21). Exposure to 3 μg/ml purified C9 without incubation (**Figure 5E**, 0 min +C9) did not induce shedding of C9^+^ EV but lead to strong C9^+^ EV release after incubation at 37°C (**Figure 6E**). The addition of purified C9 significantly increase C9^+^ EV shedding over depleted serum alone at 30 minutes (**Figure 6E**). While all three cell lines showed the same pattern across the four treatment conditions, different levels of C9^+^ EV shedding were observed. FLO-1 cells shed significantly more C9^+^ EVs than BAR-T or CP-B cell lines with, or without additional C9 (**Figure 6E**, statistical evaluations below graph). While few comparisons reached statistical significance for the C5b-9^+^ EV data, several comparisons were close to the p<0.05 cut-off (**Figure 6F**).

## Discussion

Here, we developed and applied two novel techniques to show that the local activation of complement pathway leads to C9 deposition and C5b-9/MAC formation on the epithelial component of BE and EAC, and EV shedding of C9 and C5b-9. Through analysis of tissue samples, we identified increasing C5b-9 staining along the stages of EAC development, which correspond with our previous report of increasing C9 staining. Our results support previous publications implicating lysosomes in the internalization of MAC (16). However, our investigation of C5b-9 formation and fate in BE and EAC cell lines revealed heterogeneity, which was also evident by within-group variability in the tissue staining. Emission of C9 and C5b-9 in EVs was first described as a mechanism of complement resistance in the 1980s (27, 28). In a human erythroleukemic cell line K562, mortalin (GRP75) promotes C5b-9 shedding in vesicles (29), while blocking EV release by mortalin knockdown or treatment with antagonist peptides restored complement mediated cytotoxicity in K562 and two breast cancer cells lines (30). Interestingly, C9^+^ EVs were recently suggested to mediate inflammation and neurotoxicity (31, 32), and it is intriguing to speculate that shedding of C9^+^ EV by BE and EAC cells prolongs the esophageal inflammation initiated by chronic gastroesophageal reflux and contributes to EAC progression.

As C9 transcript was reported to be increased in a rat model of gastro-esophageal reflux (11), we considered the possibility that reflux induces epithelial expression of C9. However, we did not detect a significant increase in C9 transcript relative to BE. Using cell culture of BE and EAC cell lines, we were unable to detect C9 transcript or released C9 protein prior to or following repeated bile acid exposure (data not shown) although it is possible that longer treatment or other bile acid composition is required. Hence, we found no evidence that the C9 staining observed in the esophageal tissues came from the esophageal epithelium or cancer cells, and therefore is likely deposited on esophageal tissue from a different source, possibly from the increased C9 in circulation (7). The non-significant increase in C9 transcript may originate from the immune infiltrate into the tissue, which varies between patients (33), and may have prognostic relevance (34). Indeed, we observed C9 staining in immune infiltrates in EAC tissue (6, 7), however, the immune cell type and detection of C9 transcript remain to be confirmed. While the low C9 transcript induction in EAC agrees with a recent datamining analysis in multiple cancers (1), the fact that C9 and terminal complement pathway are activated in EAC caution against relying solely on transcriptome data.

Regardless of the source of the C9, immunofluorescence microscopy on cell lines clearly demonstrates constitutive formation and fate of MAC on BE and EAC cells upon exposure to C9-depleted serum plus sublytic C9. This dose is relevant as sublytic complement attack has been reported to induce a range of changes, including protection against lysis by subsequent complement activation (35, 36), and inducing proliferation (37), promoting tumor growth and survival and avoiding cell death (16, 38, 39). A stimulus to induce a higher level of complement activation may reveal further cell-type or disease stage differences in the rate or magnitude of MAC formation, cellular localization, or clearance. It is also possible that MAC-triggered EV-shedding may induce changes to other cells (40), and the fate of these released EVs is not yet known.

While the new SERS EV assay allowed sensitive quantitation of C9^+^ EVs in cell conditioned media, several limitations are acknowledged. Firstly, the lower response for C5b-9^+^ EVs likely limited the statistical significance of the data. Based on the similar trends observed for C9^+^ and C5b-9^+^ EVs, it is tempting to speculate that both antibodies detect the same MAC-containing EVs albeit with different efficiency. This possibility remains to be formally confirmed through immuno-labelling electron microscopy studies. Secondly, we were not able to measure total EV in this study due to the large volume of cell culture medium required for total EV analysis. Hence, the possibility that a high level of C5b-9^+^ EVs shedding purely reflects a high rate of EV release remains to be investigated. Thirdly, we cannot exclude autocrine uptake of EVs by the BE/EAC cells during 30 minutes incubation time, therefore, the quantitative EV data should be considered a combination of release and uptake. Finally, the three antibodies used (CD9, CD63 and CD81) capture generic EVs (41) including ectosomes (bud from cell surface) and exosomes (endosomally-derived) (26). Future investigations are required to further characterize C9^+^ and C5b-9^+^ EVs in BE and EAC.

Our original biomarker discovery finding was that certain serum C9 glycoforms were increased in EAC (6, 7). As we performed denaturation of serum in the first step, it is possible that the particular glycoforms are shed on EVs, and potentially shed by the EAC cells. This possibility remains to be investigated. In addition, other components and regulatory proteins (42) of the complement cascade that may impact the formation of MAC are not investigated here. For example, C1q has been detected in BE and EAC (13), has both pro- and anti-tumorigenic qualities (43), and may have effects independent of classical complement activation pathways (15). The methods used here focus on the terminal complement component in MAC formation due to the raised circulating C9 previously identified (7), and the specific outcomes of MAC formation in cancer affecting tumor growth, cell death, and migration (44).

In conclusion, we show an increased terminal complement activation *via* C5b-9 formation through the stages of EAC development, which is independent of local C9 transcript change. Cultured BE and EAC cell lines respond to stimulation by sublytic C9 with C5b-9 formation and EV shedding without apparent proliferative or cytotoxic effects. With the clear demonstration of terminal complement activity in BE and EAC, future work should determine whether and how terminal complement activation plays a role in EAC development.

## Data Availability Statement

The original contributions presented in the study are included in the article/**Supplementary Material**. Further inquiries can be directed to the corresponding author.

## Ethics Statement

The studies involving human participants were reviewed and approved by QIMR Berghofer Medical Research Institute Human Research Ethics Committee (P2352). Written informed consent for participation was not required for this study in accordance with the national legislation and the institutional requirements.

## Author Contributions

CK, JW, MH, and AS conceived and designed the analysis. CK, YC, JW, RR, SB, and AL collected the data. AL and CW developed the staining methodologies. IB classified and scored the TMA. RK, KS, AW, and MT performed the EV analysis. SB and AB performed transcript assessment. WZ, GE, NC, BM, and AB provided expert analysis. CK and MH performed the analysis and wrote the paper. All authors contributed to the article and approved the submitted version.

## Funding

Translational Research Institute Spore Grant (MH); National Health and Medical Research Council APP1173669 (AW); Australian Research Council Discovery Project DP210103151 (AW, MT).

## Conflict of Interest

The authors declare that the research was conducted in the absence of any commercial or financial relationships that could be construed as a potential conflict of interest.

## Publisher’s Note

All claims expressed in this article are solely those of the authors and do not necessarily represent those of their affiliated organizations, or those of the publisher, the editors and the reviewers. Any product that may be evaluated in this article, or claim that may be made by its manufacturer, is not guaranteed or endorsed by the publisher.
