# Supplementary material for: C5b-9 Membrane Attack Complex Formation and Extracellular Vesicle Shedding in Barrett’s Esophagus and Esophageal Adenocarcinoma
Source: Front Immunol. 2022 Mar 8;13:842023. doi: 10.3389/fimmu.2022.842023 (PMC8957096; doi:10.3389/fimmu.2022.842023)
Supplement: Supplementary file 1 [file DataSheet_1.pdf]

C5b-9 membrane attack complex formation and extracellular vesicle shedding in Barrett's Esophagus and esophageal adenocarcinoma

Cathryn M. Kolka[1], Julie Webster[1], Ailin Lepletier[1], Clay Winterford[1], Ian Brown[2], Renee S. Richards[1], Wioleta M. Zelek[6], Yilang Cao[1], Ramlah Khamis[3], Karthik B. Shanmugasundaram[3], Alain Wuethrich[3], Matt Trau[3,4], Sandra Brosda[5], Andrew Barbour[5], Alok K. Shah[1], Guy D. Eslick[7], Nicholas J. Clemons[8], B. Paul Morgan[6], Michelle M. Hill[1*]

Supplementary Material

1. Supplemental Methods **Page 2**

2. Supplemental Figures and Tables

Table S1. Pixel-based colocalization analysis of C9 and C5b-9 in esophageal tissues. Page 3

Table S2. C5b-9 puncta quantification in C9-treated BE and EAC cell lines, relating to Figure 3. Page 3

Figure S1. Validation of sequential staining of C9 and C5b-9. Page 4

Figure S2. C9 mRNA transcript levels in normal, BE and EAC tissue transcriptome data. Page 5

Figure S3. Images used for quantification of C5b-9 puncta in BE and EAC cell lines. Page 6

Figure S4. Figure S4. Z-stacks highlighting cellular localization of C5b-9 in Barrett's esophagus and esophageal adenocarcinoma cell lines after exposure to purified C9 in culture. Page 11

Figure S5. Lack of C9 protein in conditioned media of unstimulated BE/EAC cell lines. Page 12

Supplemental methods:

Western Blot (Figure S5)

Transient transfection of His-Tagged C9 in HEK293 cells was conducted essentially as previously reported (7, 21). To generate conditioned media, the panel of BE and EAC cell lines as well as transfected and control HEK293 cells were washed in PBS and incubated in serum-free media with 0.1% bovine serum albumin overnight. Conditioned media were collected and the protein concentration determined using BCA assay kit (Thermo Fisher). To prepare proteins for Western blotting, 50 ug of the BE and EAC cell line conditioned media, and 2.5 ug of the HEK293 cell line conditioned media were precipitated using 100% Trichloroacetic acid. After incubation on ice for 30 min and centrifugation in 4°C for 20 min, the protein pellets were dissolved in Invitrogen™ Novex™ 4X Bolt™ LDS Sample Buffer (Invitrogen) with 10% 1 M Dithiothreitol DTT. Protein samples were heated at 95°C for 10 min and then resolved on a 10% sodium dodecyl sulphate–polyacrylamide gel electrophoresis gel, followed by wet-transfer to Immobilon®-FL PVDF membrane (Merk Millipore). After checking protein transfer by REVERT™ Total Protein Stain (LI-COR), the membrane was destained by REVERT Reversal Solution (0.1M sodium hydroxide, 30% (v/v) methanol) and blocked in PBS/5% BSA for 10 min. The membrane was incubated with mouse anti-C9 (Abcam #17931) monoclonal antibody diluted 1:1500 in PBS/5% BSA/0.1% Tween-20 at in 4°C overnight. After washing three times in PBS/0.1% Tween-20 for 10 minutes each, the membrane was incubated with the secondary antibodies of anti-rabbit 800 (Sigma) at 1:6000 dilution in foil covered tubes for 1 hour. After washing in PBS/0.1% Tween-20 three times for 10 min each, the membrane was visualized using Odyssey® CLx (LI-COR) and Image Studio software.

2. Supplementary Tables and Figures

		% C9 positivity in C5b-9				% C5b-9 Positivity in C9			
	**n**	**average**	**se**	**min**	**max**	**average**	**se**	**min**	**max**
**NSE**	**12**	**15.7%**	**4.2%**	**0.6%**	**47.8%**	**11.8%**	**3.8%**	**0.4%**	**51.5%**
**BE**	**12**	**12.7%**	**5.9%**	**0.2%**	**65.5%**	**7.5%**	**1.6%**	**0.3%**	**18.1%**
**LGD**	**13**	**15.9%**	**4.9%**	**0.4%**	**53.5%**	**11.8%**	**3.3%**	**0.6%**	**42.4%**
**HGD**	**20**	**17.6%**	**3.8%**	**0.1%**	**52.9%**	**11.1%**	**2.3%**	**0.6%**	**39.2%**
**HGD+IEC**	**14**	**23.7%**	**3.9%**	**0.0%**	**44.9%**	**8.5%**	**2.1%**	**0.0%**	**24.5%**

Table S1. Pixel-based colocalization analysis of C9 and C5b-9 in esophageal tissues. Multiplex immunofluorescent staining for C9 and C5b-9 was conducted on esophageal tissue microarray. The percent area positive for C9 in the areas positive for C5b-9, and for C5b-9 in C9 positive areas, was calculated using InForm Advanced Image Analysis software for each tissue phenotype. There was no significant difference between groups (One way ANOVA, p=0.5474 for C9 in C5b-9, p=0.7298 for C5b-9 in C9).

# Cells	UNTR	DEPL	0	30	75min
**BarT**	31	34	39	36	46
**CPA**	19	23	29	25	33
**CPB**	20	33	16	24	60
**CPD**	4	22	9	75	164
**FLO-1**	16	15	28	21	22
**OE33**		11	54	39	32
**# Puncta**	**UNTR**	**DEPL**	**0**	**30**	**75**
**BarT**	0	0	4	7	36
**CPA**	19	2	5	42	128
**CPB**	15	137	10	302	1429
**CPD**	0	2		81	225
**FLO-1**	3	6	23	20	719
**OE33**		0	0	0	7

Table S2. C5b-9 puncta quantification in C9-treated BE and EAC cell lines, relating to Figure 3. Images were counted for cell nuclei (DAPI) and C5b-9 puncta using ImageJ, with thresholding of images, followed by analysis of particles, using a particle size above 10pixels^2 for C5b-9 puncta. Images are in Figure S3, final quantification shown in Figure 3B.

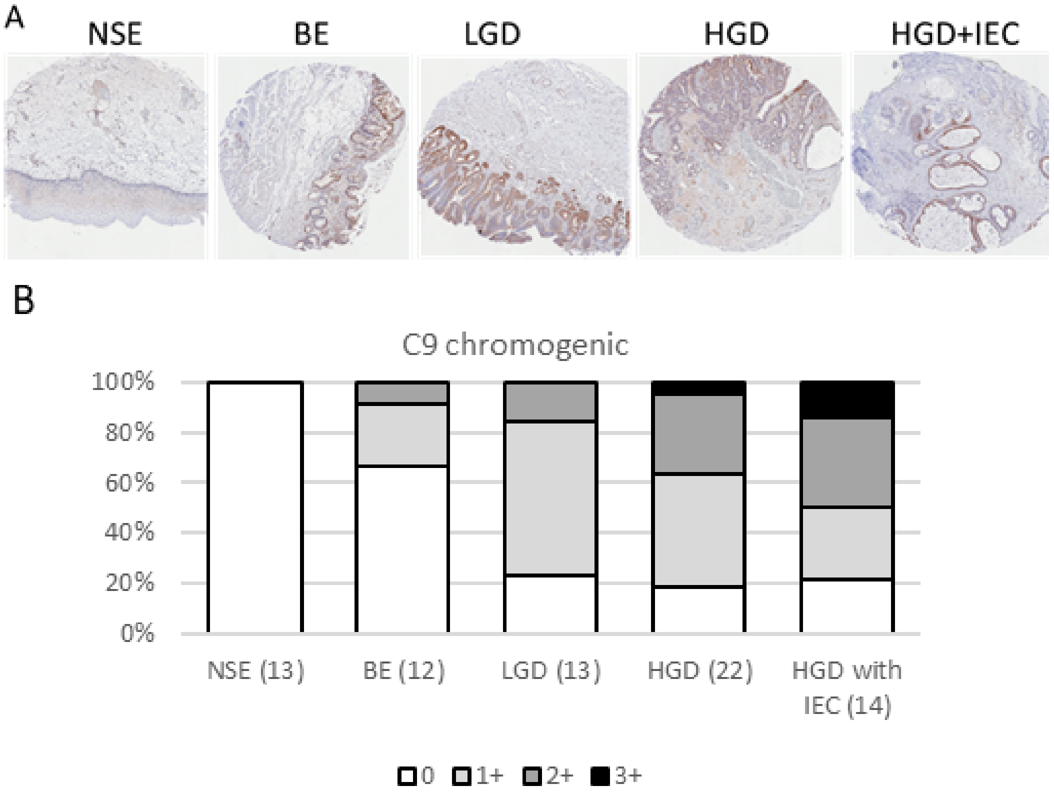

Figure S1. **Validation of sequential staining of C9 and C5b-9.** Representative chromogenic (A) stains for C9 show comparable staining patterns on separate slices from the same section as those represented in Figure 2. B) Higher C9 chromogenic staining was detected in later stages of EAC progression: scoring for C9 chromogenic staining showed significant effect likelihood ratio test with $p<0.0001$, and followed similar trends but provided scores that were not identical to immunohistofluorescence data in Figure 1.

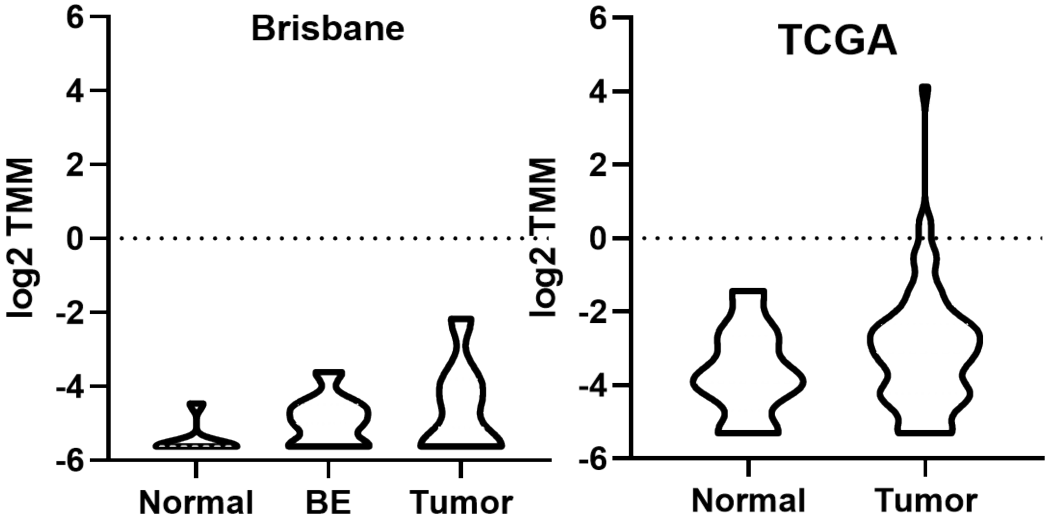

Figure S2. C9 mRNA transcript levels in normal, BE and EAC tissue transcriptome data. No statistically significant change in C9 mRNA was detected in two cohorts assessed by RNAseq.

.

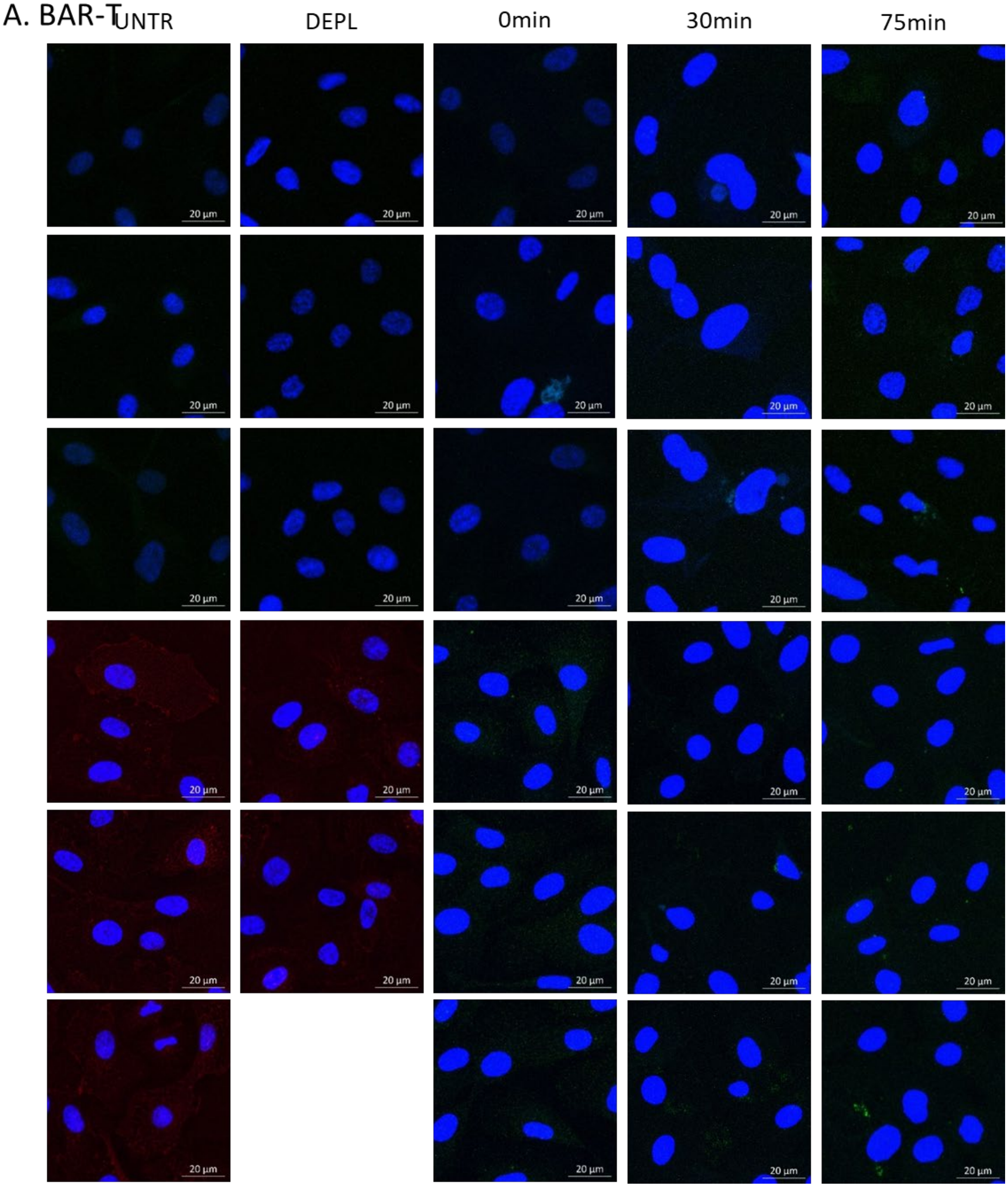

Figure S3. Images used for quantification of C5b-9 puncta in BE and EAC cell lines. BAR-T (A), CP-A (B), CP-B (C), CP-D (D), FLO-1 (E), and OE-33 (F) were imaged for DAPI (blue) and C5b-9 (green) after exposure to media plus BSA for 4 hours (untr) to remove complement components remaining in the media containing HI FBS, then 30minutes of appropriate media with C9-depleted serum (DEPL), or 0, 30, and 75 minutes of appropriate media with C9-depleted serum supplemented with 3µg/ml of immunopurified C9. Quantification of these is shown in Figure 3B.

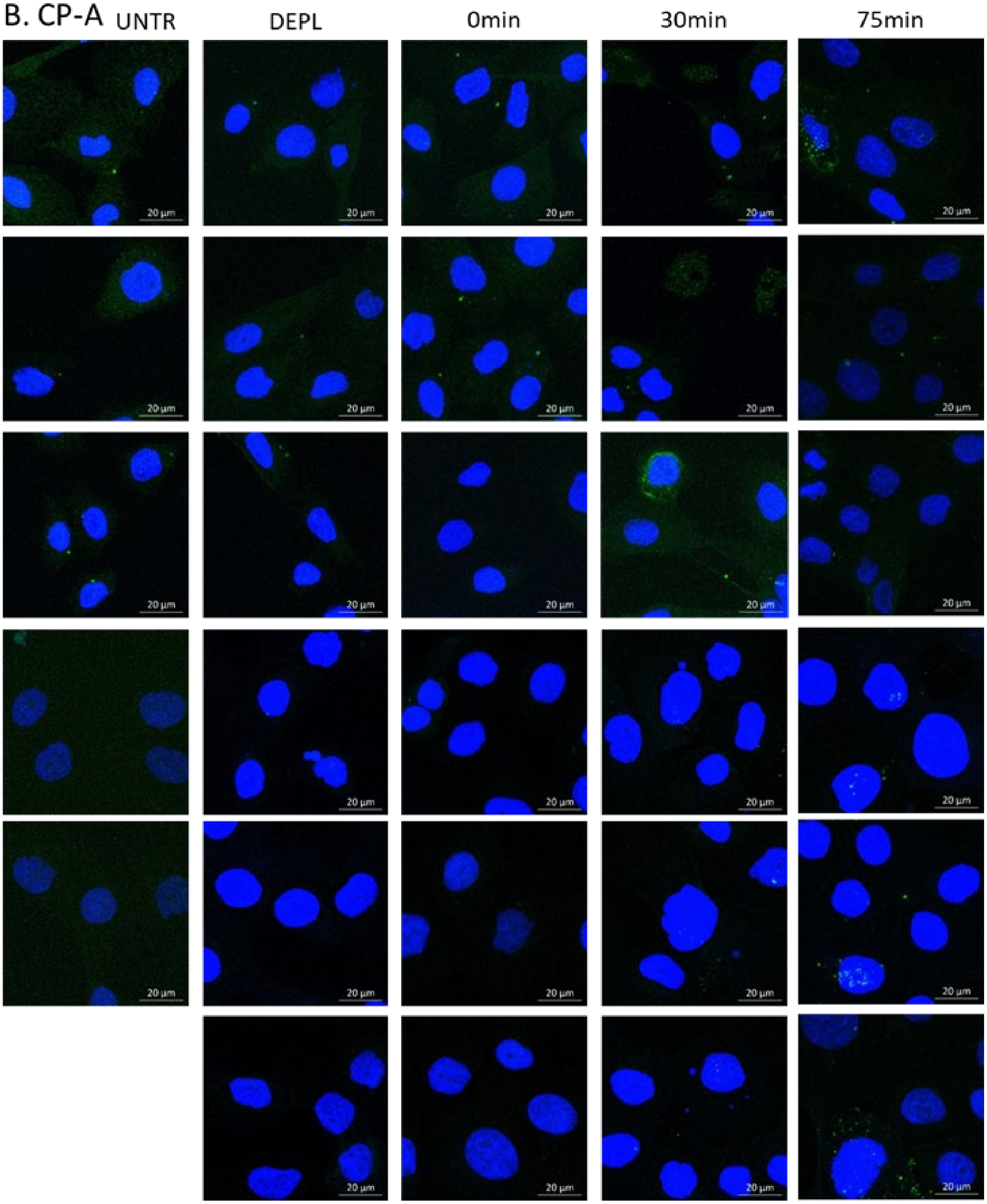
B. CP-A
UNTR
DEPL
0min
30min
75min
20 µm

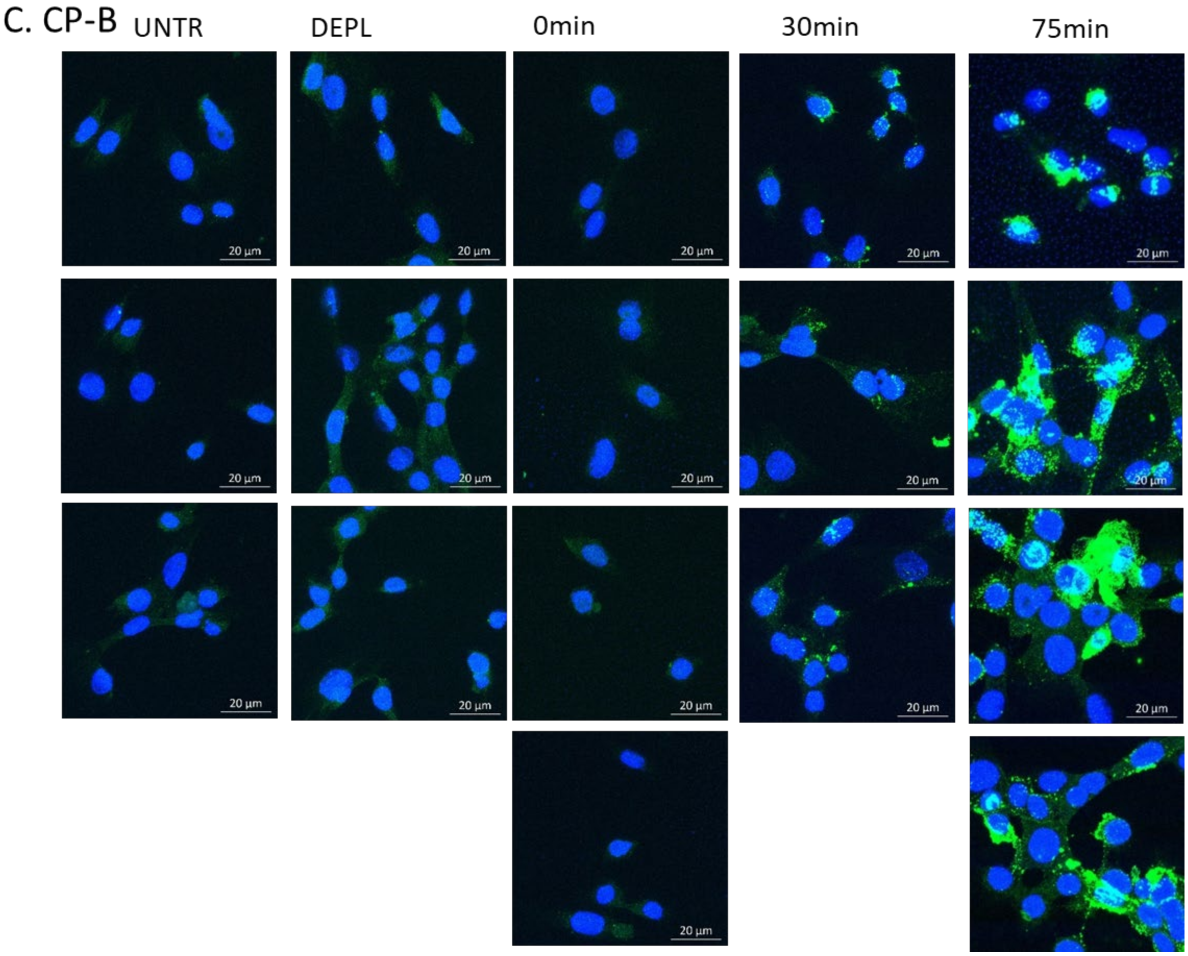
C. CP-B
UNTR
DEPL
0min
30min
75min
20 µm

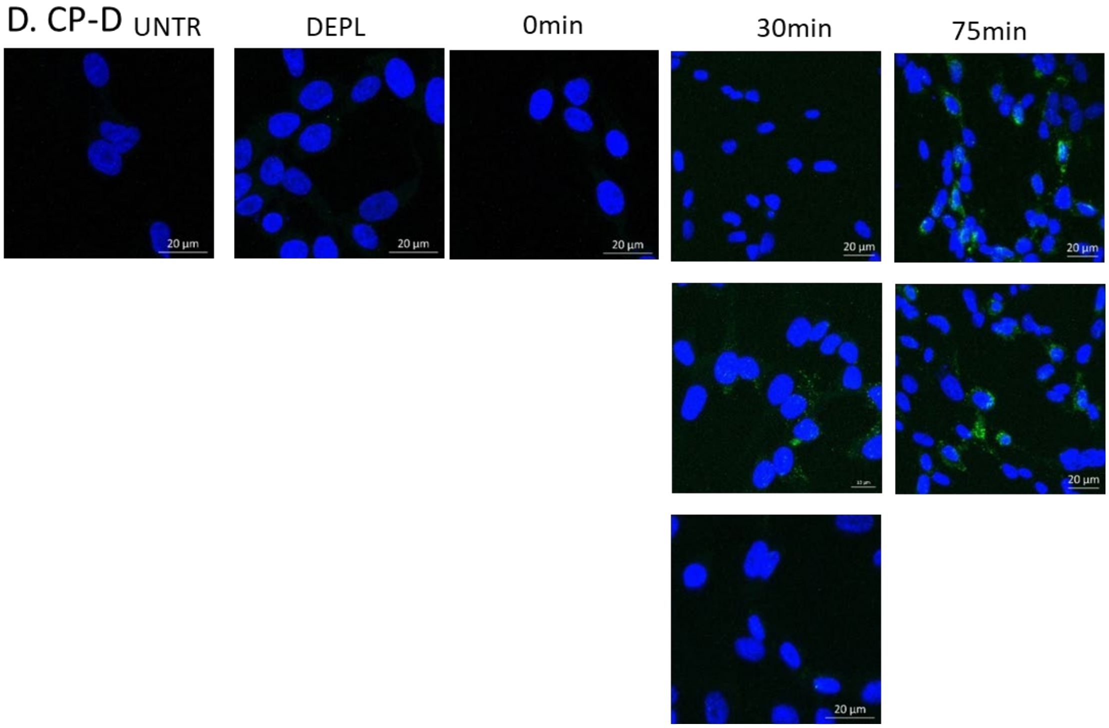
D. CP-D
UNTR
DEPL
0min
30min
75min
20 µm

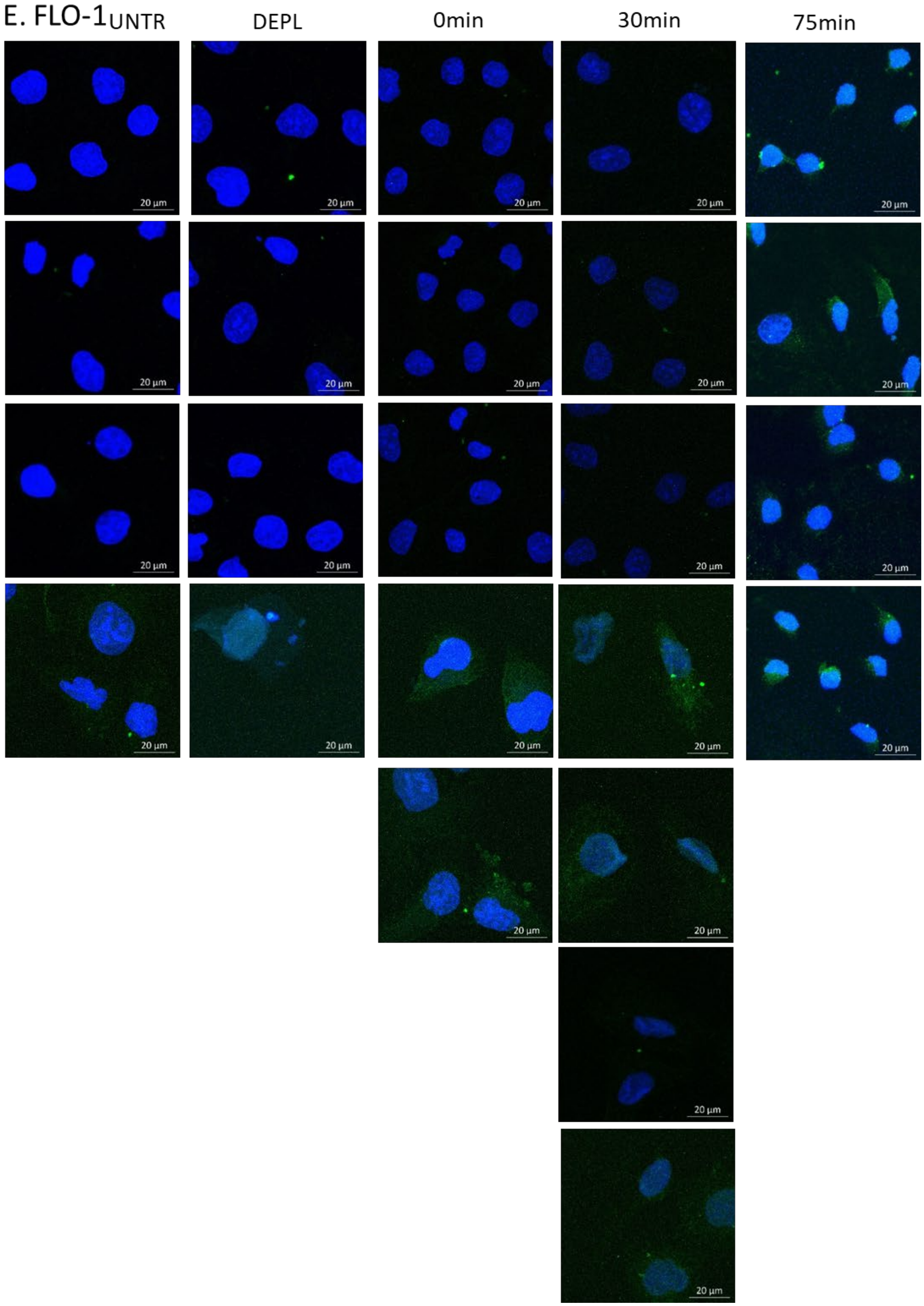
E. FLO-1UNTR
DEPL
0min
30min
75min
20 µm

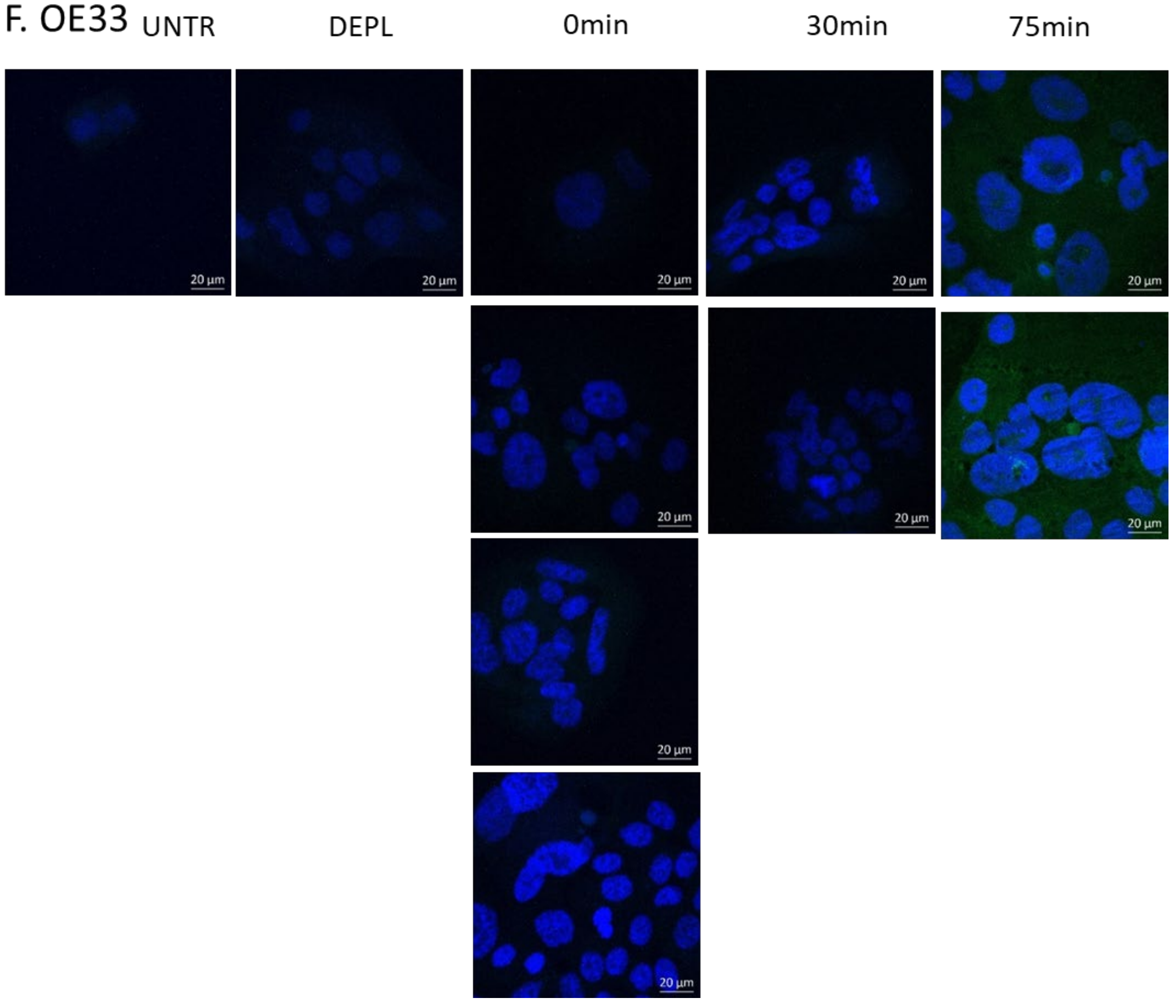
F. OE33
UNTR
DEPL
0min
30min
75min
20 µm

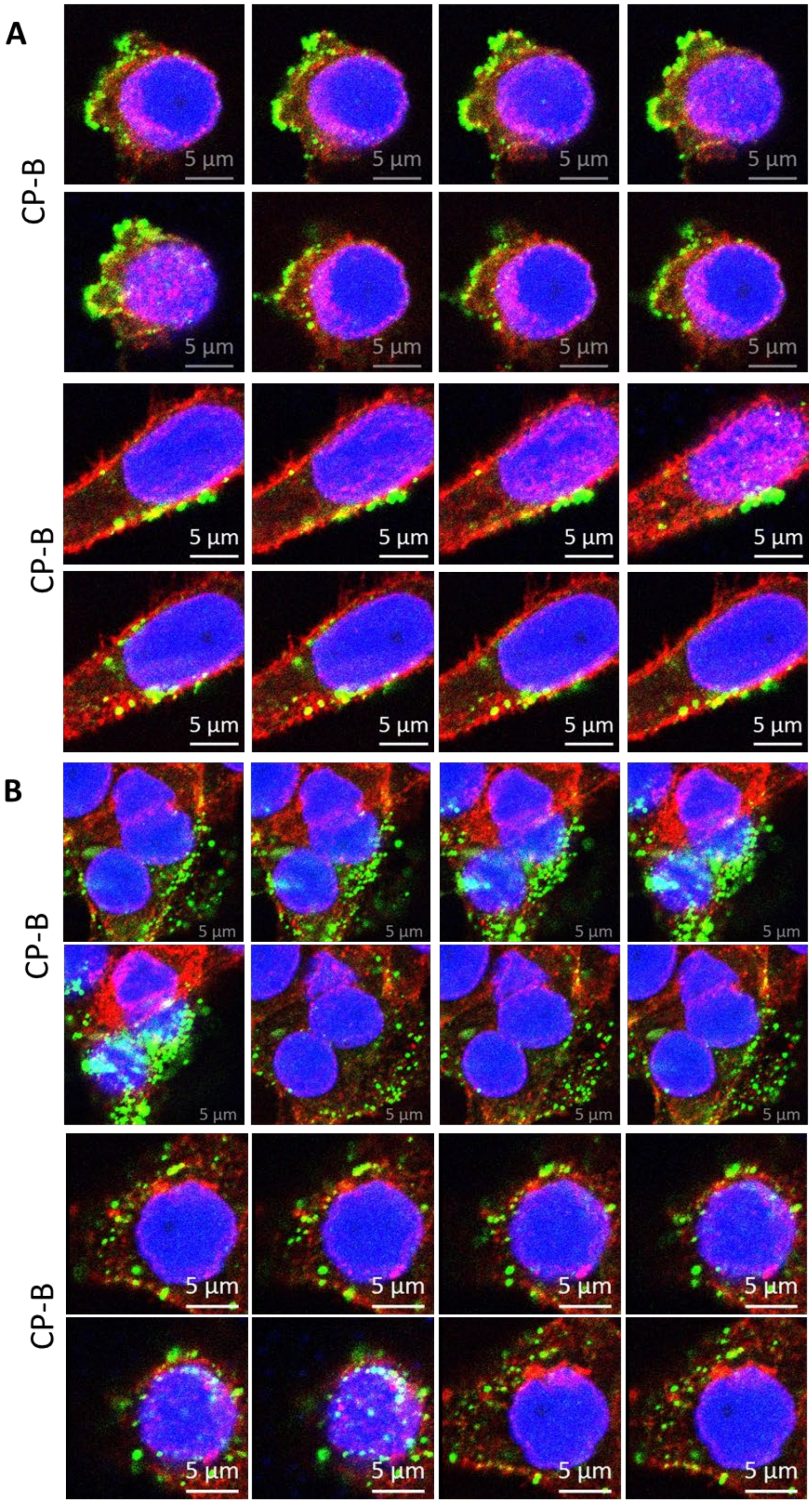
A
CP-B
CP-B
B
CP-B
CP-B
5 µm

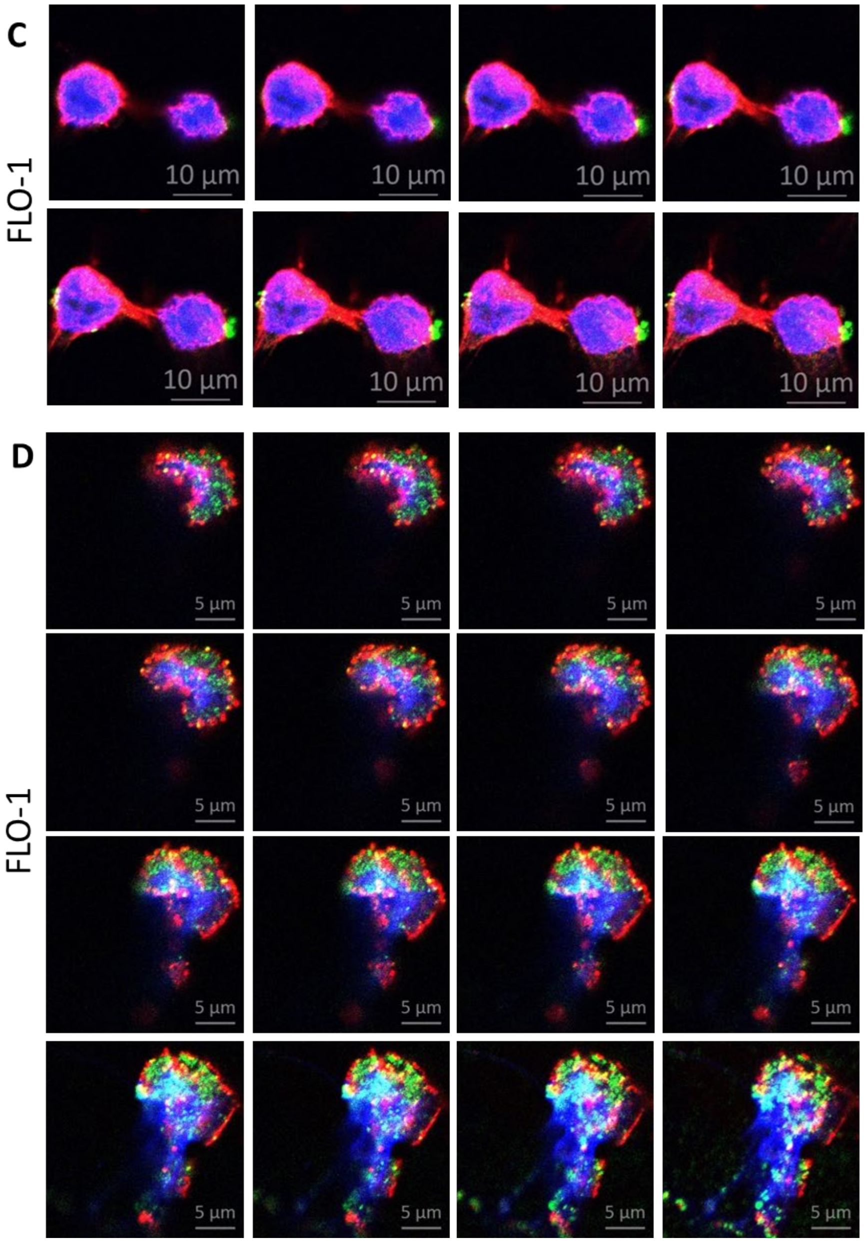

Figure S4. Z-stacks highlighting cellular localization of C5b-9 in Barrett's esophagus and esophageal adenocarcinoma cell lines after exposure to purified C9 in culture. After 75 minutes of C9 exposure of CP-B (A, B) or FLO-1 (C, D) as described for Figure 3, co-staining of C5b-9 (green) was conducted with wheat germ agglutinin (WGA, red) to visualise the plasma membrane. The nucleus was stained with DAPI (blue). C5b-9 was observed at the cell surface (A, C), as well as intracellularly (B,D). Yellow color in the combined images indicates colocalization between the red and green signals.

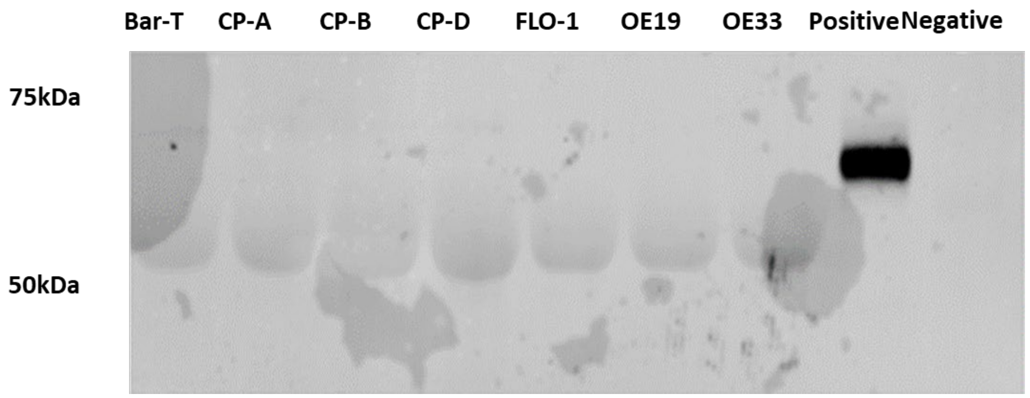

Figure S5. Lack of C9 protein in conditioned media of unstimulated BE/EAC cell lines. Conditioned media from the panel of BE and EAC cell lines were collected, and 50 µg of protein prepared for Western blotting with anti-C9 antibody. Positive control is conditioned media HEK293 cells transiently expressing His-tagged C9. Negative control is conditioned media from untransfected HEK293 cells (Webster et al, 2021). C9 was only detected in the positive control.
